# The Association between Vitamin D Insufficiency and Nonalcoholic Fatty Liver Disease: A Population-Based Study

**DOI:** 10.3390/nu9080806

**Published:** 2017-07-27

**Authors:** Yeonjung Ha, Seong Gyu Hwang, Kyu Sung Rim

**Affiliations:** Department of Hepatology, CHA Bundang Medical Center, CHA University, Seongnam-si 13496, Gyeonggi-do, Korea; sghwang@cha.ac.kr (S.G.H.); ksrimmd@cha.ac.kr (K.S.R.)

**Keywords:** diabetes, ergocalciferol, hepatic steatosis, obesity

## Abstract

Previous studies have shown inconsistent results regarding the association between vitamin D insufficiency and nonalcoholic fatty liver disease (NAFLD). We attempted to demonstrate this relationship using population-based data. Vitamin D insufficiency was defined as a 25(OH)D level ≤20 ng/mL. Hepatic steatosis index was calculated to define NAFLD. Significant fibrosis was assessed using Body mass index, AST/ALT Ratio, Diabetes (BARD) score. Logistic regression analyses were performed to determine the relationship between vitamin D insufficiency and NAFLD. Among 1812 participants, 409 (22.6%) had NAFLD. Patients with nonalcoholic fatty liver disease were more likely to be male (56.7%), had higher body mass index (28.1 kg/m^2^), and had more metabolic syndrome (57.2%). The proportion of vitamin D insufficiency did not differ between NAFLD and non-NAFLD (77.5% vs. 77.4%). Logistic regression analyses showed that BMI, diabetes, and triglyceride level were significantly associated with NAFLD, whereas vitamin D insufficiency was not related. Subgroup analyses involving non-obese participants, male participants, and participants without metabolic syndrome showed similar results. The BARD score and the proportion of significant fibrosis by BARD score did not differ according to vitamin D status. Vitamin D insufficiency was not associated with the presence of NAFLD as assessed by validated noninvasive prediction models.

## 1. Introduction

It is well known that vitamin D plays an important role in maintaining bone health by regulating calcium and phosphorus metabolism [1]. However, recent studies have demonstrated that vitamin D also has extraskeletal effects involved in various health problems [2]. For example, patients with low vitamin D levels are associated with a higher risk of diabetes, cardiovascular disease, cancer, and several autoimmune diseases [2].

In the context of the liver, Barchetta et al. reported the wide presence of vitamin D receptors in the liver and its inverse correlation with the severity of inflammation in patients with viral or metabolic hepatitis, thereby suggesting that vitamin D would influence the progression of liver diseases [3]. A later experimental study supported this hypothesis by showing that stellate cell activation was inhibited by vitamin D receptor ligands [4]. Furthermore, several clinical studies also demonstrated that vitamin D insufficiency was associated with disease progression and/or poor outcomes in patients with chronic liver disease [5].

Specifically, regarding nonalcoholic fatty liver disease (NAFLD) among chronic liver diseases, previous studies have largely reported that vitamin D insufficiency was associated with the presence [6,7,8,9] or severity [10,11] of NAFLD. However, some other studies reported quite contradictory results [12,13], and one experimental study even argued that vitamin D insufficiency alleviated accumulation of liver fat [14].

In reality, vitamin D level can be falsely decreased in obese individuals, who are more likely to have NAFLD themselves, as vitamin D is stored in the adipose tissue. Thus, determining vitamin D insufficiency as a factor related to NAFLD is not simple. In addition, the abovementioned studies mostly involved relatively small number of patients or a specific population (i.e., those with diabetes).

Therefore, we investigated the association between vitamin D insufficiency and the presence of NAFLD in a large number of population-based samples. We also stratified the samples according to the well-known risk factors for NAFLD (e.g., obesity and metabolic syndrome) to clearly determine the role of vitamin D insufficiency.

## 2. Materials and Methods

### 2.1. Participants

This study was based on the data obtained from the Sixth Korea National Health and Nutrition Examination Survey (KNHANES VI), a nationally representative cross-sectional survey that gives estimates about the health and nutritional status of the civilian, noninstitutional population in Korea. The KNHANES has been conducted periodically by the Division of Chronic Disease Surveillance, Korea Centers for Disease Control and Prevention (CDC), since 1998, and the KNHANES VI consists of surveys done from 2013 to 2015. In this study, we identified adult subjects aged >18 years who participated in the KNHANES VI in the second year (2014) and who have been checked for 25(OH)D concentrations.

Demographic characteristics and the amount of alcohol consumption based on health interviews were identified. Anthropometric characteristics, including height, weight, and waist circumference, were measured by physical examinations in a mobile center. Concomitant health problems such as hypertension, diabetes, chronic viral hepatitis, chronic kidney disease, and any type of malignancy were checked by health interviews, physical examinations, and biochemical tests. Aspartate aminotransferase (AST), alanine aminotransferase (ALT), fasting glucose, and lipid profiles were also identified. Metabolic syndrome was diagnosed according to the Asian-modified National Cholesterol Education Program-Adult Treatment Panel III criteria [15].

All the participants in this survey signed an informed consent form.

### 2.2. Serum 25(OH)D Measurement

Blood samples of individual participants were collected, refrigerated, and shipped in cold storage to Neodin Medical Institute in Seoul, Korea. Serum 25(OH)D levels were assayed using a radioimmunoassay kit (DiaSorin, Stillwater, MN, USA) within 24 h.

Vitamin D insufficiency was defined as serum 25(OH)D level <20 ng/mL based on the guideline of Food and Nutrition Board, Institute of Medicine [16,17] and multiple studies evaluating the effect of vitamin D on extraskeletal health [18,19,20,21]. The information about the date and season of serum 25(OH)D measurement was provided under the permission of Korea CDC, and 25(OH)D levels were adjusted based on the season of blood draw.

### 2.3. Definition of NAFLD and Significant Fibrosis

For defining NAFLD, the hepatic steatosis index (HSI) was calculated as follows: 8 × ALT/AST ratio + body mass index (BMI) + 2 (if diabetic) + 2 (if female). The HSI cutoff point for NAFLD is 36. This prediction tool was previously validated in >10,000 Korean subjects [22].

For defining significant fibrosis, the Body Mass Index, AST/ALT Ratio, Diabetes (BARD) score was calculated as follows: 1 (if BMI > 28) + 2 (if AST/ALT ratio > 0.8) + 1 (if diabetic) [23]. If the calculated BARD score is 2 points or higher, it is regarded as significant fibrosis. The BARD score was calculated only for participants with NAFLD.

### 2.4. Statistical Analysis

Data were presented as mean ± standard deviation (SD) for continuous variables and number and percentage for categorical variables. The relationships between NAFLD status and continuous variables were assessed by Student’s *t*-tests or Wilcoxon rank-sum tests. The relationships between NAFLD status and categorical variables were analyzed by chi-squared or Fisher’s exact tests, depending on the distribution of the variable.

The association between the baseline characteristics, including vitamin D status, and the presence of NAFLD was determined using univariable and multivariable logistic regression analyses. Subsequent subgroup analyses involving non-obese participants, male participants, and participants without metabolic syndrome, respectively, were performed.

All reported *p*-values were two-sided, and *p*-values < 0.05 were considered statistically significant. All analyses were performed using SPSS statistical package (SPSS version 20.0 for Windows; SPSS Inc., Chicago, IL, USA).

## 3. Results

### 3.1. Characteristics of the Study Participants

**Figure 1:**
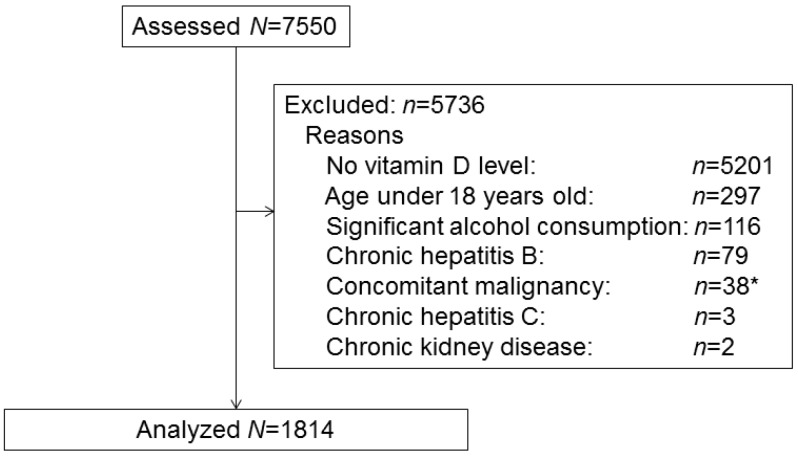
Flow diagram of subjects included in the cross-sectional study. * Thyroid cancer: *n* = 17; stomach cancer: *n* = 10; cervical cancer: *n* = 2; head and neck cancer: *n* = 2; bladder cancer: *n* = 2; breast cancer: *n* = 1; lung cancer: *n* = 1; rectal cancer: *n* = 1; endometrial cancer: *n* = 1; lymphoma: *n* = 1.

A total of 7550 participants were identified in the KNHANES VI database in 2014. Among them, 5201 participants were excluded due to lack of serum 25(OH)D levels. In addition, participants aged ≤18 years (*n* = 297), and those who had significant alcohol consumption (*n* = 116), chronic hepatitis B (*n* = 79), concomitant malignancies within five years of survey (*n* = 38), chronic hepatitis C (*n* = 3), or chronic kidney disease (*n* = 2) were excluded from the analysis. The amount of alcohol consumption was fully evaluated in the database, and a daily intake of more than 40 g of alcohol in men and 2 g in women was defined as significant alcohol consumption [24,25]. Therefore, the final study sample consisted of 1814 subjects (Figure 1).

In two of the 1814 participants, HSI cannot be calculated due to lack of relevant data. The remaining 1812 participants were divided into NAFLD vs. non-NAFLD groups according to the cutoff point of 36. Their baseline characteristics are shown in Table 1. There were significantly more males than females in the NAFLD group (56.7% vs. 44.5%, *p* < 0.001). Diabetes (22.5% vs. 4.0%, *p* < 0.001), hypertension (39.4% vs. 17.7%, *p* < 0.001), and metabolic syndrome (57.2% vs. 16.5%, *p* < 0.001) were more prevalent in the NAFLD group. Participants with NAFLD had higher BMI and waist circumference. Transaminase levels were also significantly higher in NAFLD subjects, although the mean absolute values were within normal ranges. Fasting glucose level and non-high-density lipoprotein-cholesterol lipid profiles were also higher in NAFLD subjects. However, vitamin D insufficiency, defined as 25(OH)D level <20 ng/mL, did not significantly differ between the two groups (77.5% vs. 77.4%, *p* = 1.00).

### 3.2. Factors Associated with NAFLD

The results obtained from the univariable and multivariable logistic regression analyses are presented in Table 2. The univariable analysis revealed that subjects with higher BMI, male gender, higher waist circumference, diabetes, hypertension, metabolic syndrome, higher triglyceride and lipid levels, and lower high density lipoprotein (HDL) cholesterol levels were associated with NAFLD. However, vitamin D insufficiency did not significantly correlate with the presence of NAFLD. The multivariable analysis (backward elimination) included the abovementioned variables, except for waist circumference and metabolic syndrome. Waist circumference highly correlated with BMI and metabolic syndrome composed of each variable that was entered into the multivariable analyses, i.e., diabetes, hypertension, triglyceride level, and HDL-cholesterol level; hence, we excluded these two variables to prevent multicollinearity problems. In the multivariable analysis, higher BMI (OR = 2.52, *p* < 0.001), diabetes (OR = 7.90, *p* < 0.001), and triglyceride levels (OR = 1.09, *p* = 0.005) were independently associated with NAFLD.

### 3.3. Subgroup Analyses

#### 3.3.1. Participants without Obesity

The same logistic regression analyses involving participants without obesity revealed that higher BMI (OR = 2.74, *p* < 0.001) and diabetes (OR = 10.78, *p* < 0.001) were associated with NAFLD (Table S1). Again, no significant association was observed between vitamin D insufficiency and NAFLD (crude OR = 0.75, *p* = 0.37). Baseline characteristics of this subgroup are provided in Table S2.

#### 3.3.2. Participants of Male Gender

In male participants, vitamin D insufficiency was found to be significantly associated with NAFLD in the univariable analysis, with an OR of 1.46 (95% confidence interval [CI], 1.01–2.11, *p* = 0.043). When age, BMI, diabetes, hypertension, triglyceride, total cholesterol, and HDL-cholesterol levels were added as covariates, the multivariable logistic regression analysis showed that younger age (OR = 0.95, *p* < 0.001), higher BMI (OR = 2.32, *p* < 0.001), diabetes (OR = 7.21, *p* < 0.001), and low HDL cholesterol levels (OR = 0.70, *p* = 0.005) were significantly correlated (Supplementary Table S3). Baseline characteristics of this subgroup are provided in Supplementary Table S4.

#### 3.3.3. Participants without Metabolic Syndrome

In participants without metabolic syndrome, male gender, higher BMI and waist circumference, diabetes, hypertension, higher triglyceride and total cholesterol levels, and lower HDL-cholesterol levels were found as factors associated with the presence of NAFLD using univariable analysis (Supplementary Table S5). Vitamin D insufficiency was not associated with NAFLD (crude OR = 1.19, *p* = 0.40). The factors associated with NAFLD in the multivariable analysis were higher BMI (OR = 2.54, *p* < 0.001), diabetes (OR = 5.60, *p* = 0.010), higher total cholesterol (OR = 1.63, *p* = 0.007), and lower HDL-cholesterol levels (OR = 0.77, *p* = 0.026). Baseline characteristics of this subgroup are provided in Supplementary Table S6.

### 3.4. Association of Vitamin D Insufficiency with Significant Fibrosis in Participants with NAFLD

**Figure 2:**
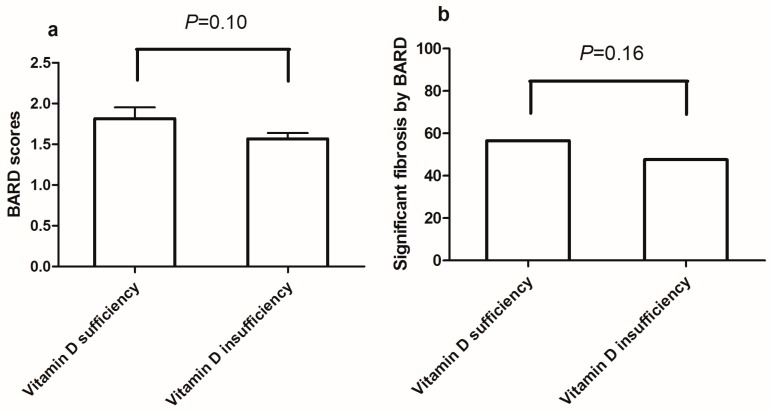
(**a**) BARD scores vs. vitamin D status; (**b**) significant fibrosis defined by BARD score vs. vitamin D status.

Comparison of the BARD score between vitamin D sufficient and insufficient subjects in the NAFLD group revealed no significant difference (mean = 1.82, standard deviation [SD] = 1.32 in vitamin D sufficient subjects vs. mean = 1.57, SD = 1.25 in vitamin D insufficient subjects; *p* = 0.10; Figure 2a). After applying the cutoff point of 2, a proportion of subjects with significant fibrosis did not demonstrate any significant difference between vitamin D sufficiency (56.5%) and insufficiency (47.6%) (*p* = 0.16, Figure 2b).

## 4. Discussion

This national survey–based cross-sectional study demonstrates that vitamin D insufficiency is not associated with the presence of NAFLD in the general population, as well as in a specific subpopulation that has less risk factor for NAFLD (e.g., subjects without obesity or metabolic syndrome). Significant fibrosis determined by the BARD score in NAFLD subjects also did not show any difference according to vitamin D status.

Vitamin D insufficiency has been a subject of attention for several researchers from various clinical backgrounds. For example, previous studies have suggested that vitamin D insufficiency was associated with cancer [18,26], cardiovascular disease [2,27], neuropsychiatric disease [26,28], and even general mortality [21]. With regard to hepatic disease, especially NAFLD, early studies showed that vitamin D insufficiency had an association with the presence, severity, and/or progression of NAFLD [6,7,8,9,11]. Some experimental studies have provided the possible mechanism involved in this phenomenon [3,10].

However, more recent studies reported quite contrary results; for example, Bril et al. demonstrated in their recent study that vitamin D insufficiency was not associated with the amount of liver fat accumulation or the severity of NASH [12]. In addition, a study by Patel et al. also showed no difference or correlation between vitamin D status and the presence or severity of NAFLD [29]. One experimental study even reported that vitamin D supplementation induced progression of NAFLD in a high fat diet–induced NAFLD mouse model [14]. Finally, a recent randomized controlled trial performed in Italy investigated the effect of oral vitamin D supplementation in NAFLD patients, only to find that vitamin D supplementation did not have any beneficial effect on hepatic steatosis and metabolic or cardiovascular parameters [30].

In addition, when we search through the previous studies, the data are rather limited for generalizability because most of the studies involved small number of patients [5,13] or a specific population that already has risk factors for NAFLD, e.g., diabetes or obesity [13]. Therefore, we attempted to elucidate the association between vitamin D insufficiency and NAFLD in a large number of nationally representative samples. As a national survey essentially provides only basic tests, we had no choice but to utilize HSI, a well-validated prediction model, to define NAFLD in this cohort. Therefore, we carried out a few subgroup analyses along with the original overall analysis to possibly overcome the limitations obtained in the process of defining NAFLD rather indirectly and found that vitamin D insufficiency was not associated with the presence of NAFLD.

Meanwhile, we considered a 25(OH) D level of <20 ng/mL as a vitamin D insufficiency. In fact, there are quite a few criteria that evaluate vitamin D status. Studies and guidelines focusing on the skeletal effects of vitamin D usually classify its level into three categories as follows: sufficiency, insufficiency, and deficiency [31]. However, studies investigating the extraskeletal complications of suboptimal 25(OH)D levels consider 20 ng/mL as a cutoff point, similar to studies of various solid organ malignancies, cardiovascular diseases, and several infections [32,33]; thus, we adopted this value as well. In addition, as the Endocrine Society recommends vitamin D supplementation when the level is ≤20 ng/mL in the general situation, this cutoff would be more relevant in clinical practice, rather than a higher value (30 ng/mL) [31].

Based on this cutoff point, vitamin D insufficiency was not associated with the presence of NAFLD; rather, diabetes was found to be consistently correlated. Therefore, we can suggest that patients with diabetes should be a target for liver assessment, especially when they have other risk factors such as obesity and hypertriglyceridemia, as shown in our results. These patients may not need to be checked for 25(OH)D concentrations just in terms of NAFLD.

In addition, even in subjects without overt metabolic syndrome, individual components were associated with NAFLD, whereas vitamin D status again was not. Higher BMI was still important, although the patients were not obese by definition. If the patients have only one or two components of metabolic syndrome, but are not yet diagnosed with the condition, they are likely to have NAFLD already. First, in terms of BMI, several studies demonstrated that higher BMI was associated with NAFLD even in non-obese subjects [34,35]. In those patients, losing weight as much as possible might not be desirable; however, we do not know the optimal cutoff of BMI for NAFLD development and progression. Moreover, changes in body composition such as sarcopenia might have been implicated in BMI measurement and pathogenesis of NAFLD. Further studies are needed to determine the optimal cutoff point for BMI for NAFLD screening and treatment. In addition, we might consider one-time screening of NAFLD with liver enzyme and/or hepatic ultrasound when there is more than one metabolic risk factor, because each of glucose and lipid profiles are associated with NAFLD even in the absence of metabolic syndrome.

Meanwhile, in terms of hepatic fibrosis, vitamin D status again was not associated with the presence of significant fibrosis in NAFLD patients. Therefore, if NAFLD was diagnosed by one-time assessment (screening) in patients with more than one risk factor, it would not be necessary to check, supplement, or follow-up their vitamin D status.

Finally, age is a reasonably complicated factor. Increasing age generally appears to be associated with the prevalence and severity of NAFLD [36,37]; however, several studies have suggested that younger age correlated with high transaminase levels or ultrasound-defined NAFLD in general or specific population (e.g., patients with metabolic syndrome) [38,39]. In our subgroup analysis involving only male participants, younger age was slightly associated with the presence of NAFLD. This result can be partly explained by the phenomenon of increasing obesity and metabolic risk factor in young males in Korea [40]. Another study performed in Korea also showed that the prevalence of ultrasound-defined NAFLD was much higher in men than in women, particularly in younger individuals (aged 20–49 years), whereas the prevalence was quite similar in both genders after 50 years of age [41]. Further studies are needed to determine whether age has a gender-specific role in the development of NAFLD.

The strengths of this study include its large sample size consisting of nationally representative samples. By utilizing this dataset, we can analyze the data of healthy individuals and are not restricted to patients with certain risk factors such as obesity. In addition, specific subgroup analyses comprising patients without traditional risk factors for NAFLD were performed to determine the effect of 25(OH)D concentrations more rigorously. After literature search, we set the cutoff point for vitamin D insufficiency as 20 ng/mL, which might better represent the extraskeletal effect of vitamin D than the bone-related effect. The information on the date of serum 25(OH)D measurement was fully available, thus appropriate seasonal adjustments were possible. Results of this study were consistent regardless of the features of patients, whether they have metabolic risk factors, significant fibrosis or not.

The study limitations include the fact that NAFLD and significant fibrosis were diagnosed by prediction models and not by a gold standard, liver biopsy. There was also no supplementary information, sometimes useful, for NAFLD diagnosis, such as abdominal ultrasonography. However, it is an inherent problem while utilizing a nationally representative sample, as performing liver biopsy and/or ultrasonography for all the individuals is neither reasonable nor ethical. Second, the cutoff point for vitamin D insufficiency in NAFLD needs to be validated in future prospective trials because the result from the association study is not the definitive one.

In conclusion, vitamin D insufficiency was not associated with the presence of NAFLD and significant fibrosis as assessed by prediction models that are based on noninvasive demographic and laboratory variables. Rather, traditional risk factors such as higher BMI and diabetes correlated with the presence of NAFLD, even in patients without obesity or overt metabolic syndrome. Thus, routine measurement of 25(OH)D concentrations does not provide additional information for the diagnosis of NAFLD and does not appear to have a role in the management of patients. For individuals with more than one traditional risk factor or higher BMI, one-time screening with liver enzymes and/or hepatic ultrasound might be helpful to identify NAFLD. For appropriate screening, the proper cutoff points for risk factors and the optimal method should be clarified in the future.

## Figures and Tables

**Table 1:** Baseline characteristics of the participants *.

Variables	NAFLD ^†^ (*n* = 409)	Non-NAFLD (*n* = 1403)	*p*-Value
Age, year	44.8 ± 14.4	43.3 ± 15.2	0.08
Male gender, *n* (%)	232 (56.7)	625 (44.5)	<0.001
BMI, kg/m^2^	28.1 ± 3.0	22.5 ± 2.5	<0.001
Waist circumference, cm	91.4 ± 8.5	77.4 ± 8.4	<0.001
Diabetes, *n* (%) ^‡^	90 (22.7)	55 (4.0)	<0.001
Hypertension, *n* (%) ^§^	159 (39.4)	247 (17.7)	<0.001
Metabolic syndrome, *n* (%) ^||^	234 (57.2)	231 (16.5)	<0.001
Season of blood draw, *n* (%)	-	-	-
Spring	106 (25.9)	359 (25.6)	0.06
Summer	90 (22.0)	393 (28.0)	
Autumn	109 (26.7)	360 (25.7)	
Winter	104 (25.4)	291 (20.7)	
AST, IU/L	25.7 ± 13.4	20.1 ± 6.5	<0.001
ALT, IU/L	37.1 ± 30.0	16.6 ± 7.8	<0.001
Fasting glucose, mg/dL	109.0 ± 31.4	94.5 ± 14.4	<0.001
Triglyceride, mg/dL	188.1 ± 134.3	120.1 ± 101.6	<0.001
Total cholesterol, mg/dL	194.2 ± 36.2	184.7 ± 32.8	<0.001
HDL-cholesterol, mg/dL	47.3 ± 10.4	54.3 ± 12.0	<0.001
25(OH)D, ng/mL	16.2 ± 6.2	16.2 ± 6.5	0.95
Vitamin D insufficiency, *n* (%)	317 (77.5)	1086 (77.4)	1.00

* Values are mean ± standard deviation. ^†^ Defined as hepatic steatosis index >36. Assessment was not possible in two patients due to lack of relevant information. ^‡^ Relevant record was not available in 34 participants. ^§^ Relevant record was not available in 12 participants. ^||^ Assessment was not possible in three patients due to lack of relevant information. NAFLD, nonalcoholic fatty liver disease; BMI, body mass index; AST, aspartate aminotransferase; ALT, alanine aminotransferase; HDL-cholesterol, high-density lipoprotein-cholesterol.

**Table 2:** Univariable and multivariable logistic regression analyses of baseline factors for the presence of nonalcoholic fatty liver disease *.

Variable	Univariable Analysis		Multivariable Analysis	
	OR (95% CI)	*p*-value	OR (95% CI)	*p*-value
Age, per 1 year	1.01 (1.00–1.01)	0.08	-	-
Male gender	1.63 (1.31–2.04)	<0.001	0.80 (0.55–1.17)	0.24
BMI, per 1 kg/m^2^	2.48 (2.26–2.73)	<0.001	2.52 (2.27–2.80)	<0.001
WC, per 1 cm	1.23 (1.20–1.26)	<0.001	-	-
Diabetes	2.66 (2.23–3.19)	<0.001	7.90 (4.62–13.51)	<0.001
Hypertension	1.74 (1.54–1.96)	<0.001	0.76 (0.52–1.12)	0.17
Metabolic syndrome	6.77 (5.32–8.61)	<0.001	-	-
TG, per 50 mg/dL	1.27 (1.21–1.34)	<0.001	1.09 (1.03–1.17)	0.005
Total cholesterol, per 50 mg/dL	1.51 (1.28–1.77)	<0.001	1.12 (0.84–1.49)	0.43
HDL-cholesterol, per 10 mg/dL	0.56 (0.51–0.63)	<0.001	0.88 (0.73–1.06)	0.19
Vitamin D insufficiency ^†^	0.97 (0.74–1.26)	0.80	-	-

* Defined as hepatic steatosis index >36. ^†^ Adjusted by season of blood draw. BMI, body mass index; WC, waist circumference; TG, triglyceride; HDL-cholesterol, high-density lipoprotein-cholesterol.

## Supplementary Materials

The following are available online at www.mdpi.com/2072-6643/9/8/806/s1, Table S1: Univariable and multivariable logistic regression analyses of baseline factors for the presence of nonalcoholic fatty liver disease in non-obese participants; Table S2: Baseline characteristics of non-obese participants; Table S3: Univariable and multivariable logistic regression analyses of baseline factors for the presence of nonalcoholic fatty liver disease in male participants; Table S4: Baseline characteristics of male participants; Table S5: Univariable and multivariable logistic regression analyses of baseline factors for the presence of nonalcoholic fatty liver disease in participants without metabolic syndrome; Table S6: Baseline characteristics of participants without metabolic syndrome.
