# Supplementary material for: The Association between Vitamin D Insufficiency and Nonalcoholic Fatty Liver Disease: A Population-Based Study
Source: Nutrients. 2017 Jul 27;9(8):806. doi: 10.3390/nu9080806 (PMC5579600; doi:10.3390/nu9080806)
Supplement: Supplementary file 1 [file nutrients-09-00806-s001.docx]

**Table S1.** Univariable and multivariable logistic regression analyses of baseline factors for the presence of nonalcoholic fatty liver disease in nonobese participants *.

| **Variable** | **Univariable Analysis** | | **Multivariable Analysis** | |
| --- | --- | --- | --- | --- |
|  | **OR (95% CI)** | ***p*-Value** | **OR (95% CI)** | ***p*-Value** |
| Age, per 1 year | 1.01 (0.99–1.02) | 0.62 |  |  |
| Male gender | 1.80 (1.03–3.14) | 0.040 | - | - |
| BMI, per 1 kg/m^2^ | 2.79 (2.08–3.74) | <0.001 | 2.74 (2.01–3.73) | <0.001 |
| WC, per 1 cm | 1.17 (1.12–1.22) | <0.001 | - | - |
| Diabetes | 3.72 (2.69–5.15) | <0.001 | 10.78 (5.20–22.35) | <0.001 |
| Hypertension | 1.53 (1.12–2.11) | 0.008 | - | - |
| Metabolic syndrome | 6.43 (3.62–11.41) | <0.001 | - | - |
| TG, per 50 mg/dL | 1.18 (1.09–1.27) | <0.001 | 1.04 (0.94–1.14) | 0.45 |
| Total cholesterol, per 50 mg/dL | 1.76 (1.19–2.60) | 0.004 | 1.36 (0.86–2.16) | 0.18 |
| HDL-cholesterol, per 10 mg/dL | 0.62 (0.48–0.81) | <0.001 | 0.93 (0.66–1.31) | 0.67 |
| Vitamin D insufficiency ^†^ | 0.75 (0.40–1.41) | 0.37 |  |  |

* Defined as hepatic steatosis index >36. ^†^ Adjusted by season of blood draw. BMI, body mass index; WC, waist circumference; TG, triglyceride; HDL-cholesterol, high-density lipoprotein-cholesterol.

**Table S2.** Baseline characteristics of non-obese participants *.

| **Variables** | **NAFLD ^†^ (*n* = 52)** | **Non-NAFLD (*n* = 1159)** | ***p*-Value** |
| --- | --- | --- | --- |
| Age, y | 44.8 ± 14.4 | 43.3 ± 15.2 | 0.62 |
| Male gender, *n* (%) | 29 (55.8) | 478 (41.2) | 0.044 |
| BMI, kg/m^2^ | 23.9 ± 1.1 | 21.7 ± 2.0 | <0.001 |
| Waist circumference, cm | 83.3 ± 6.1 | 75.5 ± 7.5 | <0.001 |
| Diabetes, *n* (%) ^‡^ | 18 (34.6) | 42 (3.7) | <0.001 |
| Hypertension, *n* (%) ^§^ | 14 (26.9) | 156 (13.5) | 0.013 |
| Metabolic syndrome, *n* (%) ^\|\|^ | 24 (46.2) | 136 (11.8) | <0.001 |
| Season of blood draw, *n* (%) |  |  | 0.20 |
| AST, IU/L | 28.1 ± 22.2 | 19.8 ± 6.2 | 0.010 |
| ALT, IU/L | 49.3 ± 52.5 | 16.3 ± 7.8 | <0.001 |
| Fasting glucose, mg/dL | 109.3 ± 30.8 | 93.7 ± 14.6 | 0.001 |
| Triglyceride, mg/dL | 185.7 ± 175.3 | 113.6 ± 98.1 | 0.005 |
| Total cholesterol, mg/dL | 196.6 ± 40.4 | 183.3 ± 32.4 | 0.023 |
| HDL-cholesterol, mg/dL | 49.1 ± 12.0 | 55.2 ± 12.0 | 0.001 |
| Vitamin D insufficiency, *n* (%) | 38 (73.1) | 907 (78.3) | 0.39 |

* Values are mean ± standard deviation. ^†^ Defined as hepatic steatosis index >36. Assessment was not possible in two patients due to lack of relevant information. ^‡^ Relevant record was not available in 18 participants. ^§^ Relevant record was not available in six participants. ^||^ Assessment was not possible in three patients due to lack of relevant information. The Korean National Health and Nutrition Examination Survey does not allow to present the specific values publicly if the number of participants in each category is less than 10. Accordingly, only *p*-value is provided and the number and percentage of participants in each season were omitted. NAFLD, nonalcoholic fatty liver disease; BMI, body mass index; AST, aspartate aminotransferase; ALT, alanine aminotransferase; HDL-cholesterol, high-density lipoprotein-cholesterol.

**Table S3.** Univariable and multivariable logistic regression analyses of baseline factors for the presence of nonalcoholic fatty liver disease in male participants *.

| **Variable** | **Univariable Analysis** | | **Multivariable Analysis** | |
| --- | --- | --- | --- | --- |
|  | **OR (95% CI)** | ***p*-Value** | **OR (95% CI)** | ***p*-Value** |
| Age, per 1 year | 0.99 (0.98–1.00) | 0.003 | 0.95 (0.94–0.97) | <0.001 |
| BMI, per 1 kg/m^2^ | 2.36 (2.08–2.67) | <0.001 | 2.32 (2.03–2.64) | <0.001 |
| WC, per 1 cm | 1.25 (1.21–1.29) | <0.001 | - | - |
| Diabetes | 3.95 (2.47–6.29) | <0.001 | 7.21 (3.44–15.08) | <0.001 |
| Hypertension | 2.14 (1.54–2.96) | <0.001 | 1.08 (0.62–1.88) | 0.78 |
| Metabolic syndrome | 5.38 (3.87–7.48) | <0.001 | - | - |
| TG, per 50 mg/dL | 1.18 (1.12–1.25) | <0.001 | 1.06 (0.98–1.14) | 0.16 |
| Total cholesterol, per 50 mg/dL | 1.36 (1.08–1.70) | 0.008 | 1.07 (0.73–1.58) | 0.73 |
| HDL-cholesterol, per 10 mg/dL | 0.54 (0.46–0.64) | <0.001 | 0.70 (0.54–0.90) | 0.005 |
| Vitamin D insufficiency ^†^ | 1.46 (1.01–2.11) | 0.043 | 1.35 (0.78–2.34) | 0.28 |

* Defined as hepatic steatosis index >36. ^†^ Adjusted by season of blood draw. BMI, body mass index; WC, waist circumference; TG, triglyceride; HDL-cholesterol, high-density lipoprotein-cholesterol.

**Table S4.** Baseline characteristics of male participants *.

| **Variables** | **NAFLD ^†^ (*n* = 232)** | **Non-NAFLD (*n* = 625)** | ***p*-Value** |
| --- | --- | --- | --- |
| Age, y | 41.1 ± 13.9 | 44.5 ± 15.2 | 0.002 |
| BMI, kg/m^2^ | 28.0 ± 2.8 | 23.0 ± 2.4 | <0.001 |
| Waist circumference, cm | 93.5 ± 8.0 | 81.3 ± 7.4 | <0.001 |
| Diabetes, *n* (%) ^‡^ | 45 (20.4) | 37 (6.1) | <0.001 |
| Hypertension, *n* (%) ^§^ | 88 (37.9) | 138 (22.2) | <0.001 |
| Metabolic syndrome, *n* (%) ^\|\|^ | 125 (53.9) | 111 (17.8) | <0.001 |
| Season of blood draw, *n* (%) |  |  |  |
| Spring | 59 (25.4) | 164 (26.2) | 0.17 |
| Summer | 53 (22.8) | 185 (29.6) |  |
| Autumn | 64 (27.6) | 153 (24.5) |  |
| Winter | 56 (24.1) | 123 (19.7) |  |
| AST, IU/L | 27.4 ± 12.3 | 21.8 ± 7.2 | <0.001 |
| ALT, IU/L | 43.2 ± 27.1 | 19.5 ± 8.3 | <0.001 |
| Fasting glucose, mg/dL | 108.1 ± 26.5 | 96.7 ± 15.9 | <0.001 |
| Triglyceride, mg/dL | 215.1 ± 150.9 | 147.3 ± 127.5 | <0.001 |
| Total cholesterol, mg/dL | 192.6 ± 37.4 | 185.7 ± 31.8 | 0.013 |
| HDL-cholesterol, mg/dL | 44.2 ± 9.5 | 50.6 ± 11.2 | <0.001 |
| Vitamin D insufficiency, *n* (%) | 182 (78.4) | 442 (70.7) | 0.025 |

* Values are mean ± standard deviation. ^†^ Defined as hepatic steatosis index >36. Assessment was not possible in two patients due to lack of relevant information. ^‡^ Relevant record was not available in 28 participants. ^§^ Relevant record was not available in four participants. ^||^ Assessment was not possible in three patients due to lack of relevant information. NAFLD, nonalcoholic fatty liver disease; BMI, body mass index; AST, aspartate aminotransferase; ALT, alanine aminotransferase; HDL-cholesterol, high-density lipoprotein-cholesterol.

**Table S5.** Univariable and multivariable logistic regression analyses of baseline factors for the presence of nonalcoholic fatty liver disease in participants without metabolic syndrome *.

| **Variable** | **Univariable Analysis** | | **Multivariable Analysis** | |
| --- | --- | --- | --- | --- |
|  | **OR (95% CI)** | ***p*-Value** | **OR (95% CI)** | ***p*-Value** |
| Age, per 1 year | 1.00 (0.99–1.01) | 0.50 |  |  |
| Male gender | 2.03 (1.46–2.81) | <0.001 | 0.94 (0.55–1.60) | 0.81 |
| BMI, per 1 kg/m^2^ | 2.57 (2.25–2.92) | <0.001 | 2.54 (2.22–2.90) | <0.001 |
| WC, per 1 cm | 1.24 (1.20–1.28) | <0.001 | - | - |
| Diabetes | 3.72 (2.69–5.15) | <0.001 | 5.60 (1.51–20.73) | 0.010 |
| Hypertension | 1.70 (1.15–2.53) | 0.008 | 0.68 (0.35–1.33) | 0.26 |
| TG, per 50 mg/dL | 1.17 (1.09–1.26) | <0.001 | 1.03 (0.92–1.15) | 0.60 |
| Total cholesterol, per 50 mg/dL | 1.62 (1.29–2.04) | <0.001 | 1.63 (1.15–2.33) | 0.007 |
| HDL-cholesterol, per 10 mg/dL | 0.65 (0.55–0.76) | <0.001 | 0.77 (0.61–0.97) | 0.026 |
| Vitamin D insufficiency ^†^ | 1.19 (0.79–1.79) | 0.40 |  |  |

* Defined as hepatic steatosis index >36. ^†^ Adjusted by season of blood draw. BMI, body mass index; WC, waist circumference; TG, triglyceride; HDL-cholesterol, high-density lipoprotein-cholesterol.

**Table S6.** Baseline characteristics of participants without metabolic syndrome *.

| **Variables** | **NAFLD ^†^ (*n* = 175)** | **Non-NAFLD (*n* = 1169)** | ***p*-Value** |
| --- | --- | --- | --- |
| Age, y | 40.0 ± 14.2 | 40.8 ± 14.7 | 0.50 |
| Male gender, *n* (%) | 107 (61.1) | 511 (43.7) | <0.001 |
| BMI, kg/m^2^ | 27.5 ± 2.6 | 22.1 ± 2.4 | <0.001 |
| Waist circumference, cm | 88.8 ± 8.3 | 75.9 ± 7.8 | <0.001 |
| Diabetes, *n* (%) ^‡^ | 9 (5.4) | 22 (1.9) | 0.012 |
| Hypertension, *n* (%) ^§^ | 26 (15.0) | 117 (10.0) | 0.06 |
| Season of blood draw, *n* (%) |  |  |  |
| Spring | 45 (25.7) | 305 (26.1) | 0.19 |
| Summer | 39 (22.3) | 334 (28.6) |  |
| Autumn | 45 (25.7) | 292 (25.0) |  |
| Winter | 46 (26.3) | 238 (20.4) |  |
| AST, IU/L | 24.6 ± 13.7 | 19.7 ± 6.0 | <0.001 |
| ALT, IU/L | 37.5 ± 33.3 | 16.1 ± 7.5 | <0.001 |
| Fasting glucose, mg/dL | 97.4 ± 17.4 | 92.0 ± 10.7 | <0.001 |
| Triglyceride, mg/dL | 136.3 ± 94.8 | 102.7 ± 83.6 | <0.001 |
| Total cholesterol, mg/dL | 194.7 ± 36.6 | 183.4 ± 32.7 | <0.001 |
| HDL-cholesterol, mg/dL | 51.0 ± 10.7 | 56.0 ± 11.5 | <0.001 |
| Vitamin D insufficiency, *n* (%) | 143 (81.7) | 923 (79.0) | 0.43 |

* Values are mean ± standard deviation. ^†^ Defined as hepatic steatosis index >36. Assessment was not possible in two patients due to lack of relevant information. ^‡^ Relevant record was not available in 26 participants. ^§^ Relevant record was not available in five participants. NAFLD, nonalcoholic fatty liver disease; BMI, body mass index; AST, aspartate aminotransferase; ALT, alanine aminotransferase; HDL-cholesterol, high-density lipoprotein-cholesterol.
